# PIrates of the vacuole: phosphoinositide hijacking by T4SS intracellular pathogens

**DOI:** 10.1093/femsml/uqag032

**Published:** 2026-10-26

**Authors:** Matteo Bonazzi

**Affiliations:** IRIM, University of Montpellier, CNRS, 34293 Montpellier, France

**Keywords:** T4SS, bacterial pathogens, host cell lipids, *Coxiella*, *Legionella*, *Brucella*

## Abstract

Intracellular bacteria equipped with a Type IV Secretion Systems (T4SS), including *Legionella pneumophila, Coxiella burnetii, Brucella abortus*, and *Anaplasma/Ehrlichia* species, evolved a convergent strategy of exploiting host cell lipids at every stage of infection, from cholesterol-rich raft-mediated entry to the establishment of replicative vacuoles that evade lysosomal destruction. Indeed, these pathogens actively remodel host cell membranes by deploying T4SS effectors that bind, generate, or deplete specific lipid species, in particular phosphoinositides and cholesterol, to dictate the identity and trafficking fate of their containing vacuole. A remarkable diversity of effectors with lipid-binding or lipid-modifying activities has now been characterized across these pathogens: from the PI4P-centric Dot/Icm effector cascade of *Legionella* to the cooperating CvpB, CvpE, and Vice triad of *Coxiella*, and the phosphoinositide-binding immune evasion effector BtpA of *Brucella*. These pathogens also tap into host lipid droplets for energy and membrane precursors, modulate bioactive lipid mediators such as prostaglandins to curb immune clearance, and present atypical surface lipids to minimize pattern recognition. Advances in lipidomics, including a landmark systematic identification of novel α-helical PI-binding effector folds conserved across *Legionella* and *Coxiella*, have shed new light on these processes with unprecedented resolution. This review examines the shared and divergent lipid-subversion strategies of T4SS-bearing intracellular pathogens from an integrated molecular, cellular, and lipidomic perspective, and highlights emerging opportunities for host-directed therapeutic intervention.

## Teaser

U2OS cell expressing Lact-C2-mCherry (yellow) infected by GFP-expressing Coxiella burnetii (pink)

## Introduction

The intracellular lifestyle imposes a fundamental challenge: a replicating bacterium must secure nutrients, evade degradation, and suppress immune detection while confined within a living cell whose every compartment is subject to sophisticated surveillance. Proteins and nucleic acids have historically dominated conceptual frameworks of host–pathogen interaction, but it is now clear that lipids are equally key players. Lipids define the identity of cellular membranes, carry second-messenger information, fuel intracellular metabolism, and orchestrate immune signalling (van Meer et al. 2008, Matteis and Godi 2004); as such, they are simultaneously the primary substrate and the primary target of intracellular parasitism.

By delivering effector proteins directly into the host cytoplasm, type 4 secretion systems (T4SS) allow bacteria to reprogram host cell behaviour at multiple stages of infection with remarkable precision. The Dot/Icm shared by *Legionella pneumophila* and *Coxiella burnetii*, and the VirB T4SS of *Brucella abortus*, represent the most extensively characterized examples (Hubber and Roy 2010, Voth and Heinzen 2009, Dugelay et al. 2025), with related systems found in *Anaplasma phagocytophilum* and *Ehrlichia chaffeensis* (Rikihisa 2018). These pathogens share a strategic imperative: to transform their vacuolar compartment into a replicative niche invisible to, or actively tolerated by, host surveillance machinery. Importantly, host lipids are central to virtually every step of this transformation (Fig. 1).

**Figure 1:**
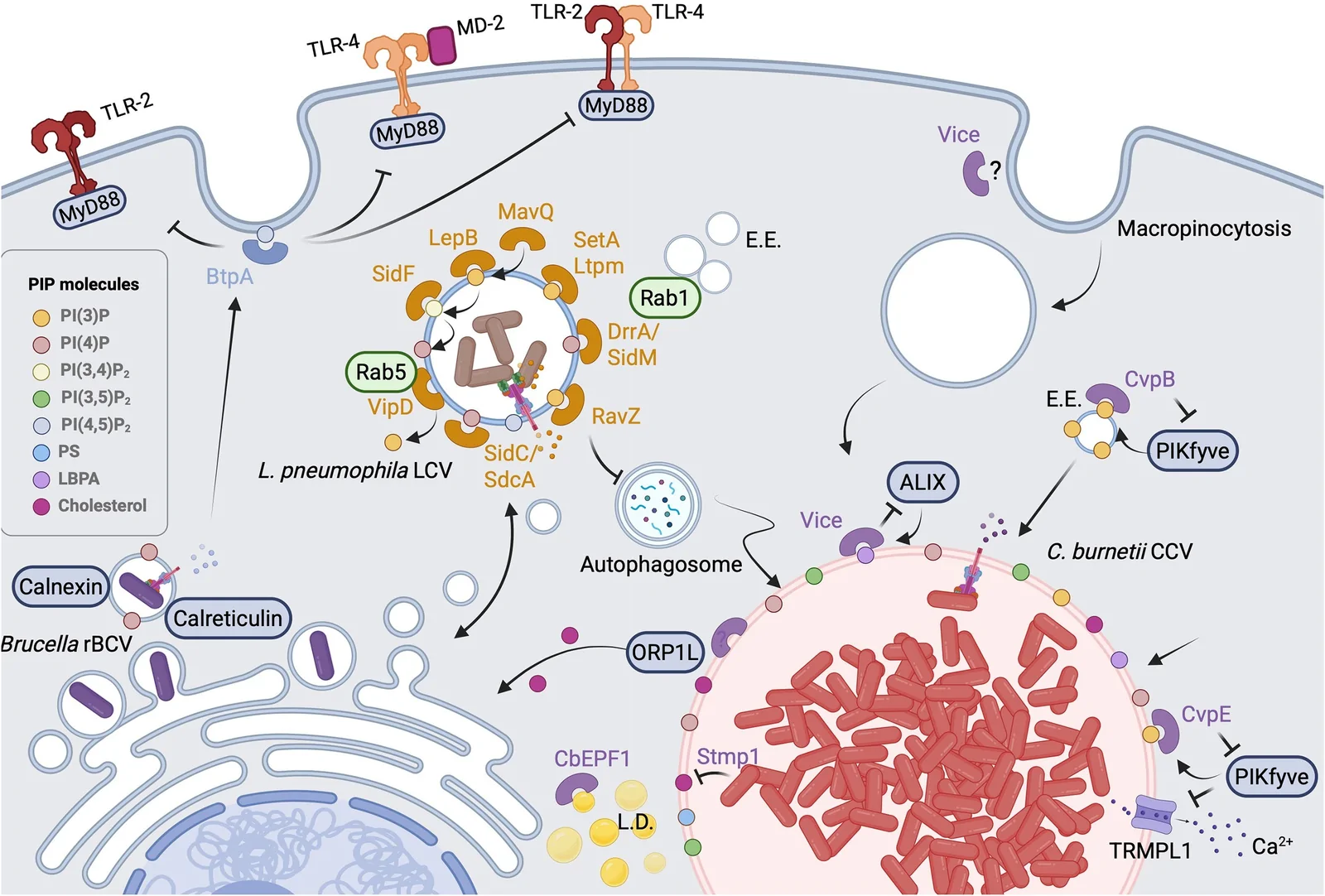
Manipulation of host lipid and membrane biology by T4SS effectors of intracellular bacterial pathogens. Schematic overview of key T4SS-translocated effector functions deployed by *L. pneumophila, Brucella* spp., and *C. burnetii* to remodel their respective pathogen-containing vacuoles (PCVs) and subvert host innate immune signalling. Coloured circles represent distinct phosphoinositide (PIP) species and lipid molecules as indicated in the legend (left). **(*Left*)** The *Brucella* replicative BCV (rBCV) intercepts the ER secretory pathway and is progressively remodelled into a compartment that is physically continuous with, and forms part of, the ER, acquiring ER markers calnexin and calreticulin. The T4SS effector BtpA binds to plasma membrane phosphoinositides and inhibits MyD88-dependent TLR signalling at the plasma membrane, dampening pro-inflammatory cytokine production. **(*Centre*)** During *L. pneumophila* infection, the *Legionella*-containing vacuole (LCV) acquires a PI(4)P-enriched identity through the concerted action of MavQ, LepB, and SidF T4SS effectors. DrrA/SidM and LepB act as a GEF–GAP pair to recruit and cycle Rab1, driving ER-derived vesicle fusion and preventing phagolysosomal maturation. SidC/SdcA tether PI(4)P-positive membranes to the LCV. VipD, a phospholipase, removes PI(3)P from early endosome (E.E.) membranes, preventing Rab5-mediated endosomal fusion. RavZ irreversibly deconjugates LC3 from autophagosomes, blocking autophagy-mediated bacterial clearance. **(*Right*)** The *C. burnetii*-containing vacuole (CCV) is a large lysosome-derived compartment enriched in PI(3)P, PI(4)P, PI(3,5)P₂, Phosphatidylserine (PS), lysobisphosphatydic acid (LBPA) and cholesterol. The T4SS effector Vice stimulates macropinocytosis to promote initial CCV expansion and impairs the membrane targeting of the ESCRT-associated protein ALIX to stabilize CCVs. The effector CvpB inhibits PIKfyve kinase activity, modulating PI(3,5)P₂ levels and promoting vacuolar homotypic fusion. CvpE similarly inhibits PIKfyve at a later stage, while also antagonizing the Ca²⁺ channel TRPML1, reducing lysosomal Ca²⁺ release. A yet unidentified effector recruits the cholesterol transporter ORP1L to CCVs to modulate cholesterol at CCVmembranes. CbEPF1 facilitates lipid droplet (L.D.)—ER—CCV interactions. The sterol modifying protein Stmp1 regulates cholesterol levels at CCV membranes. Blunt-ended arrows denote inhibitory interactions; standard arrows indicate activation, recruitment, or translocation. Abbreviations: LCV, *Legionella*-containing vacuole; rBCV, replicative *Brucella*-containing vacuole; CCV, *Coxiella*-containing vacuole; E.E., early endosome; E.R., endoplasmic reticulum; L.D., lipid droplets. Created in BioRender. Bonazzi, M. (2026) https://BioRender.com/49fymi2

The effector repertoires deployed by these pathogens to manipulate host lipids are now known to be vast. Recent systematic screens have identified more than 30 novel phosphoinositide-binding effectors in *Legionella* alone (Bolt 2025), revealing that bacterial PI-binding proteins employ compact α-helical folds structurally distinct from canonical eukaryotic lipid-binding domains. This strategy, which is conserved in *Coxiella*, points to convergent evolution of membrane-targeting mechanisms across phylogenetically distant T4SS pathogens (Bolt 2025). For *Coxiella*, at least three characterized Dot/Icm effectors (CvpB, CvpE, and Vice) target distinct aspects of PI metabolism and membrane dynamics to collectively shape the *Coxiella*-containing vacuole (CCV) (Martinez et al. 2016, Zhao et al. 2024, Bienvenu et al. 2024) (Fig. 1). In addition, *Coxiella* finely tunes CCV cholesterol levels, which are bacteriolytic when in excess (Gilk et al. 2010, Clemente et al. 2022, Mulye et al. 2017), and actively promotes host lipid droplets formation to support intracellular replication (Justis et al. 2016, Schuler et al. 2023, Angara et al. 2025) (Fig. 1). For *Brucella*, the effector BtpA (TcpB/Btp1) combines a phosphoinositide-binding N-terminal domain with a C-terminal TIR domain, linking lipid targeting to innate immune evasion in a single protein (Radhakrishnan et al. 2009, Coronas-Serna et al. 2020) (Fig. 1). More broadly, the capacity to exploit specific phosphoinositide species for subcellular targeting is not restricted to these four T4SS pathogens: a systematic survey of bacterial effector–membrane interactions identified phosphoinositide-binding activity as a recurring targeting strategy across diverse intracellular pathogens, where recognition of a specific lipid species is often sufficient to direct an effector to its functional organelle or membrane compartment (Weigele et al. 2017).

This review examines how T4SS-equipped intracellular bacteria exploit host lipid biology across three interconnected aspects of the intracellular lifestyle: the manipulation of phosphoinositide and cholesterol dynamics to establish and maintain a replicative vacuole; the acquisition and metabolism of host lipids as nutrients; and the modulation of bioactive lipid mediators to suppress immune responses. We also examine the effector proteins through which these manipulations are executed and consider how modern lipidomics is revealing the full landscape of lipid remodelling during infection. By focusing on *Legionella, Coxiella, Brucella*, and *Anaplasma/Ehrlichia*, we aim to illustrate how these pathogens have converged on a common set of lipid-centred solutions to the challenges of intracellular survival, and that this convergence identifies both fundamental principles of host/pathogen interactions and promising targets for novel therapeutic intervention.

### Lipids at the host–pathogen interface: entry and vacuole biogenesis

The first interaction between a T4SS pathogen and its host cell occurs at the plasma membrane, where lipid composition profoundly shapes both the mode and early outcome of bacterial uptake. A unifying feature is preferential association with cholesterol-rich, sphingolipid-enriched membrane microdomains during entry. The lipid environment of the entry portal directly influences the identity of the nascent vacuole and, consequently, the intracellular fate of the bacterium (Lafont and Van Der Goot 2005).

*Brucella abortus* smooth strains bind macrophage lipid rafts via scavenger receptor SR-A, using their O-polysaccharide LPS as a ligand (Kim et al. 2004). Raft-mediated entry is functionally required for productive infection as strains entering through cholesterol-rich domains establish replication permissive vacuoles, whereas rough LPS mutants failing to engage rafts are rapidly delivered to lysosomes (Kim et al. 2004). An important downstream consequence of raft engagement is the suppression of macrophage apoptosis. Indeed, SR-A signalling within rafts delivers anti-apoptotic signals that block caspase-2 activation, preserving the host cell as a viable intracellular niche (Kim et al. 2004). Consistent with this raft-dependent mechanism, pharmacological disruption of the cholesterol gradient using cholesterol-scavenging or -binding agents inhibits both internalization and intracellular replication of *Brucella* (Naroeni and Porte 2002), and ketoconazole-mediated blockade of cholesterol transport from the lysosome to the plasma membrane similarly impairs bacterial uptake and replication, with *Brucella* co-localizing with cholesterol-binding filipin shortly after entry (Watarai et al. 2002, Watarai et al. 2002). Independently of T4SS-related mechanisms, *Brucella* also deploys cyclic β-1,2-glucans that disrupt cholesterol-rich lipid rafts and are required to prevent phagosome–lysosome fusion, providing a further route by which raft cholesterol is exploited to secure the replicative niche (Bohin 2000, Arellano-Reynoso et al. 2005).

*Coxiella burnetii* also exploits cholesterol-rich microdomains during uptake, and disruption of raft integrity reduces internalization efficiency (Gilk et al. 2013). Unlike *Brucella* and *Legionella, Coxiella* ultimately requires an acidic, hydrolase-positive phagolysosomal environment for replication (Clemente et al. 2023). The lipid composition of the CCV has now been systematically mapped using fluorescent lipid-binding sensors: PI(3,5)P2 and phosphatidylserine (PS) are passively recruited to CCV membranes, while PI3P, PI4P, and lysobisphosphatidic acid (LBPA) accumulate in a T4SS-dependent manner (Bienvenu et al. 2024).

*Legionella pneumophila* enters macrophages by conventional phagocytosis but Dot/Icm translocation can occur before phagocytosis is complete (Nagai et al. 2005). In contrast to *Coxiella*, the LCV membrane is sterol-poor, reflecting its ER-derived identity. Active sterol exchange at LCV–ER membrane contact sites mediated by oxysterol-binding proteins (OSBPs) drives net cholesterol depletion from the LCV membrane, promoting vacuole remodelling (Vormittag et al. 2023). Nevertheless, host cholesterol homeostasis is required to support *L. pneumophila* intracellular replication, as pharmacological disruption of cholesterol metabolism impairs bacterial growth without affecting LCV biogenesis or maturation (Ondari et al. 2022).

*Anaplasma phagocytophilum* and *Ehrlichia chaffeensis* similarly rely on cholesterol-rich microdomains for efficient uptake (Lin and Rikihisa 2003). Both are severely auxotrophic for lipids and scavenge host cholesterol and sphingomyelin extensively from the outset, intercepting Golgi and recycling endosome vesicle traffic (Lin and Rikihisa 2003, Lin et al. 2020).

### Phosphoinositide remodelling: rewriting the vacuole’s lipid identity

Phosphoinositides (PIs) are quantitatively minor membrane lipids with major functional importance. Their specific phosphorylation state determines which cytoplasmic proteins associate with a given membrane, thereby defining organelle identity and directing vesicle traffic (Angara et al. 2025). PI3P marks early endosomes; PI(3,5)P2 characterizes late endolysosomes; PI4P marks the Golgi and ER exit sites; and PI(4,5)P2 is enriched at the plasma membrane. LBPA, although not itself a phosphoinositide, is a related anionic phospholipid that serves as a hallmark of multivesicular bodies. Normal phagosome maturation involves a sequential PI transition from PI3P through PI(3,5)P2 to lysosomal fusion. T4SS-bearing pathogens subvert this progression by rewriting the PI code on their vacuolar membranes.

#### Legionella pneumophila: a multi-effector PI4P-centric strategy

The LCV’s defining lipid feature is its enrichment in PI4P (Weber et al. 2006), which causes the LCV to be recognized by host trafficking machinery as an ER-like compartment, enabling it to intercept ER-derived vesicles and bypass endolysosomal maturation. This transformation is executed by a functionally complementary set of Dot/Icm effectors (Swart and Hilbi 2020) (Fig. 1).

Early after internalization, the effector MavQ functions as a PI3-kinase generating PI3P on the nascent phagosome (Li et al. 2021, Hsieh et al. 2021) , which serves as substrate for the PI4-kinase activity of LepB (Dong et al. 2016). LepB phosphorylates PI3P to PI(3,4)P2, and SidF subsequently acts as a PI3-phosphatase converting PI(3,4)P2 to PI4P, thereby completing a three-enzyme (MavQ, LepB, SidF) biosynthetic relay (Dong et al. 2016, Li et al. 2021) (Fig. 1). Once a nascent LCV is established, the effector DrrA (SidM) is central to PI4P-driven LCV biogenesis. Its P4M domain binds PI4P with nanomolar affinity, ensuring exclusive LCV localization (Brombacher et al. 2009). DrrA then functions as a GEF activating Rab1, which promotes docking of ER-derived vesicles at the LCV membrane (Murata et al. 2006) (Fig. 1). Finally, SidC and its paralogue SdcA independently bind PI4P, anchoring their ubiquitin ligase activities to the LCV to recruit ER tethering factors (Ragaz et al. 2008) (Fig. 1).

During infection, the phospholipase A1 activity of VipD, a Dot/Icm effector activated by Rab5 binding, protects LCVs from entering the endocytic pathway by depleting phospholipids from host endosomes (Gaspar and Machner 2014). In addition, the effectors SetA and LtpM combine C-terminal PI3P-binding domains with N-terminal glycosyltransferase activity, integrating membrane targeting with enzymatic subversion of host trafficking machinery (Jank et al. 2012, Levanova et al. 2019, Im et al. 2026). LtpD independently binds PI3P via an internal 17-kDa fragment and additionally interacts with the host enzyme IMPA1 (Harding et al. 2013). The effector LpnE, a Sel1-repeat protein, binds PI3P and is required for optimal entry and intracellular trafficking (Newton et al. 2007, Weber et al. 2009). Together, these effectors illustrate a recurring strategy in which PI3P-rich membranes serve as a spatial cue that concentrates enzymatically active effectors at the LCV membrane, ensuring precise and localized subversion of host membrane identity.

A recent systematic screen of 241 *Legionella* effectors using lipid bead pulldowns and mass spectrometry identified over 30 novel PI-binding effectors, more than doubling the known repertoire (Bolt 2025). These effectors share compact α-helical folds structurally distinct from canonical eukaryotic PH, FYVE, or PX domains. Crucially, this α-helical PI-binding module is conserved in *C. burnetii* and *Burkholderia pseudomallei*, suggesting convergent evolution of PI recognition across phylogenetically diverse intracellular pathogens (Bolt 2025).

Beyond PI manipulation, *Legionella* secretes the effector protein RavZ to disable host autophagy. RavZ contains a membrane-inserting lipid-binding helix that recognizes PE-conjugated LC3 and irreversibly cleaves the PE moiety, permanently inactivating LC3 (Choy et al. 2012). The effector LegS2 (LipA) functions as an S1P lyase degrading sphingosine-1-phosphate, simultaneously restraining autophagic capture of the LCV and dampening NF-κB-mediated inflammatory signalling through S1P receptor pathways (Degtyar et al. 2009, Abu Khweek et al. 2016).

#### Coxiella burnetii: a triad of lipid-interacting effectors shapes the CCV

Differently from *L. pneumophila, C. burnetii* allows CCVs to travel along the endocytic pathway until fusion with late endosome/lysosomes. Regardless, *Coxiella* T4SS effectors severely reshape the vacuole’s lipid identity to acquire and stabilize membranes and modulate intravacuolar acidification. Thus far, three lipid-interacting effectors (LIEs) have been identified among the repertoire of *Coxiella* T4SS substrates: CvpB, CvpE, and Vice, with distinct, non-overlapping roles in CCV biogenesis, although the precise biochemical basis of this functional distinction remains incompletely defined.

CvpB binds PI3P and PS via its N-terminal domain, localizing to early endosomes and CCV membranes (Martinez et al. 2016). At early endosomes, CvpB inhibits the PI5-kinase PIKfyve, preventing conversion of PI3P to PI(3,5)P2 (Martinez et al. 2016) (Fig. 1). The resulting PI3P enrichment drives vacuolation and clustering of early endosomes, channelling large PI3P-positive membranes to CCVs for vacuole expansion (Martinez et al. 2016). Simultaneously, PI3P accumulation on CCV membranes recruits LC3-positive membranes to CCVs, promoting homotypic CCV fusion (Martinez et al. 2016). Although early studies reported no replication defect for cvpB mutants in standard cell-monolayer infections, more recent work using lower multiplicities of infection has shown that loss of CvpB does impair intracellular replication (Steiner and Roy 2024, Bird et al. 2025). Independently of this replication phenotype, loss of CvpB produces a multivacuolar phenotype and attenuated virulence in the *Galleria mellonella* and SCID mouse infection models (Martinez et al. 2016, Schaik et al. 2017), indicating that vacuole development has *in vivo* relevance.

CvpE operates in a complementary spatial context: it binds PI3P and perturbs PIKfyve activity on lysosomes rather than early endosomes (Zhao et al. 2024). CvpE suppresses the lysosomal Ca^2+^ channel TRPML1 indirectly through its PIKfyve-inhibitory activity, preventing lysosomal tubulation and fission (Zhao et al. 2024) (Fig. 1). By blocking lysosomal fragmentation, CvpE favours CCVs expansion and bacterial replication. The distinct spatial focus of CvpB and CvpE activities (lysosomes versus early endosomes) reveals that *Coxiella* has evolved separate effectors to control PI3P metabolism at different points along the endolysosomal pathway, indicating that CvpB and CvpE act on spatially distinct PI3P pools during CCV maintenance, although whether this reflects true functional complementarity or parallel, non-redundant activities remains to be determined.

The effector protein Vice (for Vacuole-inducing *Coxiella* effector) adds a third dimension (Bienvenu et al. 2024). Vice binds multiple PIP species and interacts with membranes enriched in PS and LBPA, a hallmark lipid of multivesicular bodies, and is responsible for the enrichment of LBPA to CCV membranes (Bienvenu et al. 2024). Ectopic expression of Vice in non-infected cells triggers macropinocytosis, generating Vice-induced compartments (VICs) that closely resemble early CCVs in lipid composition (Bienvenu et al. 2024). Vice also perturbs the ESCRT machinery, possibly by perturbing ALIX interactions with LBPA, inhibiting intraluminal vesicle formation and stabilizing VICs in non-infected cells and CCVs during infection (Bienvenu et al. 2024) (Fig. 1).

*Coxiella* also recruits the host cholesterol transporter ORP1L to the CCV membrane through an as-yet-unidentified effector, forming CCV/ER membrane contact sites that facilitate cholesterol transport from the CCV to the ER (Fig. 1). This prevents the accumulation of toxic levels of cholesterol while supplying cholesterol for bacterial membrane synthesis (Justis et al. 2016). In addition, the bacterial outer membrane sterol reductase Stmp1 provides a further layer of cholesterol homeostasis at the CCV membrane (Clemente et al. 2022) (Fig. 1).

#### Anaplasma and ehrlichia: ER-derived vacuoles and obligate lipid auxotrophy

*Anaplasma phagocytophilum* and *E. chaffeensis* are severely auxotrophic for lipids, lacking genes for lipid A and peptidoglycan biosynthesis and relying instead on host-derived cholesterol, which is incorporated directly into their membranes to confer structural integrity (Lin and Rikihisa 2003). The *A. phagocytophilum* vacuole (ApV) additionally intercepts recycling endosome traffic by selectively recruiting Rab GTPases (including Rab4A, Rab10, Rab11A, Rab14, Rab22A, and Rab35) to its vacuolar membrane (Huang et al. 2010), and exploits Rab10-dependent delivery of trans-Golgi network-derived vesicles into the vacuole lumen to acquire host sphingomyelin and ceramide, which are essential for bacterial morphotype conversion and infectious progeny production (Truchan et al. 2016). The ApV also forms extensive membrane contact sites with the endoplasmic reticulum, with ER-derived vesicles delivered directly into the vacuole lumen throughout the infection cycle (Truchan et al. 2016).

### Lipid acquisition and metabolism: exploiting host stores

If establishing a replicative vacuole solves the problem of survival, securing lipids as nutrients and membrane precursors is equally essential for intracellular growth. Thus, T4SS pathogens exploit host lipid droplets, membrane cholesterol, and ER-derived phospholipids as primary nutritional sources.

*Coxiella*-infected macrophages accumulate cytosolic lipid droplets (LDs) in a T4SS-dependent manner (Fig. 1). LD lipolysis is essential for intracellular bacterial growth as pharmacological inhibition of adipose triglyceride lipase (ATGL) severely impairs *Coxiella* replication (Mulye et al. 2018). LD breakdown likely serves two purposes: supplying fatty acids to support bacterial replication, and releasing arachidonic acid for prostaglandin E2 (PGE2)-mediated immunosuppression within the macrophage (Mulye et al. 2018). The Dot/Icm effector CbEPF1 is directed by its FFAT motif to ER–lipid droplet contact sites, possibly facilitating nutrient channelling from LDs to the CCV (Angara et al. 2024). Lipid droplets serve as both the arachidonic acid reservoir and enzymatic platform for eicosanoid biosynthesis in leukocytes (Bozza and Viola 2010, Weller 2016). Emerging evidence suggests that *Coxiella* exploits this capacity to generate PGE2-mediated immunosuppression within infected macrophages (Mulye et al. 2018).

*Legionella*’s conversion of the LCV into an ER-like compartment positions it adjacent to the cell’s primary lipid synthetic hub. LCV-ER membrane contact sites, maintained by PI4P-binding effectors, enable cholesterol, phospholipids, and other ER lipids to flow into the vacuole (Vormittag et al. 2023). Oxysterol-binding proteins play a critical role at these contact sites: OSBP8 localizes to the ER and restricts *Legionella* replication, while OSBP11 localizes to the LCV membrane and promotes LCV expansion and bacterial replication (Vormittag et al. 2023). Finally, the type II-secreted enzyme PlaC possesses glycerophospholipid:cholesterol acyltransferase (GCAT), phospholipase A, and lysophospholipase A activities, transferring fatty acids from host glycerophospholipids onto cholesterol to remodel the host membrane environment. However, PlaC is dispensable for intracellular replication in macrophage models (Banerji et al. 2005).

The intimate association of BCVs with the ER enables direct metabolic integration. The Pcs PC synthase captures host choline headgroups released from phospholipid and sphingomyelin breakdown at the ER interface, synthesizing PC for the bacterial outer membrane (Comerci et al. 2006).

The severely limited lipid biosynthetic repertoires of *Anaplasma* and *Ehrlichia* make them among the most lipid-dependent intracellular pathogens, incorporating host cholesterol and sphingomyelin directly into their own membranes (Lin and Rikihisa 2003). Their absolute dependence on host lipid supply makes lipid acquisition pathways particularly attractive targets for therapeutic intervention.

### Lipid–protein interactions and T4SS effector architecture

The lipid manipulations described above are executed through direct molecular interactions between bacterial effectors and host lipid species or lipid-binding proteins. A defining architectural feature of lipid-interacting T4SS effectors is the inclusion of lipid-binding modules that direct effectors to specific membrane compartments, a targeting mechanism conferring extraordinary precision. Lipid-interacting effector proteins discussed here are listed in Table 1, together with their lipid targets and biological function.

**Table 1:** Effector proteins involved in host lipids interactions.

Pathogen	Effector	Lipid target/domain	Biological outcome	References
***Legionella pneumophila***	DrrA/SidM	PI4P—P4M domain	Anchors to LCV; recruits and activates Rab1; redirects early secretory vesicles	(Murata et al. 2006, Brombacher et al. 2009, Del Campo et al. 2014)
	SidC/SdcA	PI4P—P4C domain (four antiparallel α-helix bundle)	Anchors to LCV; recruits ER proteins; ubiquitinates host targets	(Ragaz et al. 2008, Luo et al. 2015)
	MavQ	PI (substrate)	Generates PI3P on LCV as substrate for LepB; initiates PI4P biosynthetic relay	(Li et al. 2021, Hsieh et al. 2021)
	LepB	PI3P (substrate)—N-terminal kinase domain	Advances PI4P relay; terminates Rab1 activity cycle	(Dong et al. 2016)
	SidF	PI(3,4)P2 and PI(3,4,5)P3 (substrates)	Completes LCV PI4P biosynthetic relay; establishes PI4P-rich membrane identity	Dong et al. 2016, Li et al. 2021)
	VipD	PI3P (on endosomal membranes)	Depletes PI3P from endosomal membranes; displaces EEA1 and fusion factors; renders endosomes fusion-incompetent; protects LCV from endosomal fusion	(Gaspar and Machner 2014, Lucas et al. 2014)
	RavZ	PI3P (targeting via PI3P-binding module); PE-LC3 conjugate (substrate)	Irreversibly prevents LC3 re-lipidation; blocks autophagosome maturation; evades xenophagy	(Choy et al. 2012, Horenkamp et al. 2015)
	LtpD	PI3P (internal fragment LtpD471–626)	Targets LCV and PI3P-positive endosomal compartments; modulates inositol phosphate cycle; required for efficient macrophage replication	(Harding et al. 2013)
	SetA	PI3P (activating ligand)	Glucosylates host protein substrates (TFEB)	(Jank et al. 2012, Levanova et al. 2019, 2019, Im et al. 2026)
	LtpM	PI3P—C-terminal binding domain	Targets LCV, early and late endosomes; glucosylates host protein substrates; first member of novel glucosyltransferase effector family	(Jank et al. 2012, Levanova et al. 2019, 2019, Im et al. 2026)
	LpnE	PI3P—Sel1-like repeats	Required for efficient host cell entry and intracellular trafficking	(Newton et al. 2007, Weber et al. 2009)
***Coxiella burnetii***	CvpB	PI3P and PS	Channels PI3P-positive endosomal and autophagosomal membranes to CCV; essential for CCV homotypic fusion and expansion; mutant shows multivacuolar phenotype	(Martinez et al. 2016, Schaik et al. 2017)
	CvpE	PI3P	Suppresses lysosomal tubulation and TRPML1-dependent fission; prevents lysosome fragmentation; maintains single large CCV; mutant shows replication and PV defects	(Zhao et al. 2024)
	Vice	PS and LBPA	Initiates CCV biogenesis by triggering internalization of large vacuoles; stabilizes nascent vacuoles	(Bienvenu et al. 2024)
	CbEPF1	Host lipid droplets (FFAT motif → VAP proteins on ER)	Localizes to host LDs; tethers LDs to ER; modulates host LD growth	(Angara et al. 2024)
***Brucella abortus***	BtpA	Phosphoinositides at the PM —N-terminal domain	Subverts innate immune recognition; prevents pro-inflammatory cytokine production; facilitates BCV biogenesis	(Radhakrishnan et al. 2009, Sengupta et al. 2010, Salcedo et al. 2013, Coronas-Serna et al. 2020)

#### Legionella: canonical and novel PI-binding folds

The Dot/Icm effector repertoire of *Legionella* contains the most comprehensively mapped collection of bacterial lipid-binding modules. DrrA’s P4M domain binds PI4P with nanomolar affinity (Del Campo et al. 2014). SidC independently recognizes PI4P via a distinct globular domain (Luo et al. 2015). VipD is activated by Rab5 binding, which unmasks its phospholipase A1 active site, coupling Rab GTPase sensing to phospholipid hydrolysis on endosomal membranes (Gaspar and Machner 2014, Lucas et al. 2014). LtpD contains an internal PI3P-binding fragment and additionally engages IMPA1, an enzyme in the inositol monophosphate cycle (Harding et al. 2013). The effector Lem3 functions as a dephosphocholinase that reverses AnkX-mediated phosphorylcholination of Rab1, thereby restoring Rab1 accessibility to its regulators and providing temporal control over early secretory pathway manipulation at the LCV (Tan et al. 2011).

The systematic screen revealing over 30 novel PI-binding effectors in *Legionella* has dramatically extended this picture (Bolt 2025). These effectors share compact α-helical folds structurally distinct from the canonical PH, FYVE, and PX domain families—novel bacterial solutions to the protein–lipid recognition problem (Bolt 2025). Critically, this α-helical PI-binding fold is structurally conserved in *Coxiella* and *Burkholderia* effectors, suggesting convergent evolution of membrane-targeting strategy (Bolt 2025).

RavZ illustrates a particularly elegant lipid–protein interaction: it targets PI3P-enriched, highly curved autophagosomal membranes via a PI3P-binding module and a catalytic domain helix, recognizes PE-conjugated LC3/Atg8, and irreversibly cleaves the amide bond within Atg8, rendering it unable to be re-lipidated and thereby blocking autophagy (Choy et al. 2012, Horenkamp et al. 2015). Finally, SetA and LtpM combine PI-binding with glycosyltransferase activity, modifying host proteins at PI-defined membrane locations (Levanova et al. 2019, 2019).

#### Coxiella: complementary effectors targeting distinct lipid pools

The three lipid-interacting effectors (LIEs) of *Coxiella*, CvpB, CvpE, and Vice, each employ distinct mechanisms to target distinct lipid pools (Martinez et al. 2016, Zhao et al. 2024, Bienvenu et al. 2024). CvpB and CvpE both bind PI3P and inhibit PIKfyve, but their spatial specificity differs: CvpB acts on early endosomes and CCV membranes, while CvpE targets lysosomes (Martinez et al. 2016, Zhao et al. 2024). Vice operates on a different lipid axis entirely, targeting LBPA and PS rather than PIs, and engages components of the ESCRT machinery as its protein partners (Bienvenu et al. 2024). The observation of different morphological phenotypes associated with mutations in each effector illustrates how CCVs biogenesis is finely tuned. The conservation of the α-helical PI-binding fold identified in *Legionella* in *Coxiella* effectors suggests that additional PI-binding effectors in *Coxiella* remain to be experimentally characterized (Bolt 2025).

#### Brucella: lipid-binding effectors linking membrane targeting to immune evasion

*Brucella* vacuolar development is complex and proceeds in two phases. The nascent BCV first transits through an endosomal intermediate, where moderate acidification activates VirB T4SS expression (Celli et al. 2003). VirB effectors then redirect the BCV toward the ER, ultimately remodelling it into a compartment that is physically continuous with, and forms part of, the ER, generating a replicative BCV decorated with ER markers (calnexin, calreticulin) and ER-characteristic lipids (Celli et al. 2003, Starr et al. 2012) (Fig. 1).

A notable lipid-targeting VirB effector is BtpA, which combines an N-terminal domain that binds phosphoinositides at the plasma membrane with a C-terminal TIR domain that mimics the host adaptor TIRAP (Radhakrishnan et al. 2009, Salcedo et al. 2013). Through its PI-binding domain, BtpA targets plasma membrane PIs in a manner analogous to TIRAP, promoting MAL polyubiquitination and degradation, thereby blocking TLR signalling (Sengupta et al. 2010). BtpA additionally interacts directly with the death domain of MyD88, further disrupting TLR-mediated inflammatory cascades (Salcedo et al. 2013) (Fig. 1). This dual-function architecture, lipid-targeting domain for subcellular localization, and immune-evasion domain for effector function, illustrates how *Brucella* integrates membrane targeting with downstream immunosuppression in a single effector protein.

### Bacterial structural lipids as immune camouflage: stealth LPS and PC

The lipid-dependent strategies discussed so far concern pathogen manipulation of host-derived lipids. However, the bacterial cell envelope itself is a major lipid-based determinant of host immune recognition, and T4SS intracellular pathogens have subjected their own structural lipids to striking evolutionary modifications that reduce immunostimulatory activity and enable prolonged intracellular persistence.

Both *B. abortus* and *C. burnetii* have evolved lipopolysaccharides with structurally adapted lipid A moieties leading to markedly reduced TLR4-stimulating activity and resulting in immune evasion. *Brucella* LPS is non-endotoxic, confers resistance to antimicrobial defences, and modulates host immune responses, properties that have been characterized as key virulence mechanisms enabling intracellular survival (Lapaque et al. 2005). Virulent *Coxiella* phase I LPS actively antagonizes TLR4 signalling by impairing p38α-MAPK-driven phosphorylation and blocking Vps41-HOPS-mediated trafficking of bacteria-containing vacuoles to phagolysosomes (Barry et al. 2012). For Brucella, this evasion extends beyond lipid A: a distinctive core oligosaccharide additionally shields the lipid A moiety from TLR4 recognition, and disruption of this core through mutation of *wadC* substantially increases TLR4 signalling (Conde-Álvarez et al. 2012, Guimarães et al. 2025). BtpA contributes a further, LPS-independent layer of TLR evasion through the lipid-targeting and TIR-mimicry mechanism described above (Radhakrishnan et al. 2009, Salcedo et al. 2013, Sengupta et al. 2010). Finally, *Brucella*’s PC-rich outer membrane, built using the choline-utilizing PC synthase Pcs described above, constitutes a third structural layer of immune camouflage. Accordingly, Pcs mutants display altered envelope properties and are attenuated for chronic infection in mice, indicating that PC is required for full virulence (Comerci et al. 2006, Conde-Alvarez et al. 2006).

### Emerging technologies and future directions

A recurring limitation in current studies of T4SS effector-mediated lipid manipulation is the reliance on targeted, hypothesis-driven approaches, filipin staining, fluorescent lipid sensors, pharmacological depletion, and sterol profiling, that characterize individual lipid classes rather than the global membrane environment of the pathogen vacuole. Technological advances in mass spectrometry-based lipidomics offers the means to address this gap systematically.

In related intracellular infection models, genuine untargeted lipidomics has already begun to reshape our understanding of host/pathogen lipid biology. LC-MS-based lipidomic profiling of drug-resistant Mycobacterium tuberculosis clinical isolates revealed lipidome signatures linked to antibiotic tolerance: drug-resistant strains showed shifts in inner-membrane glycerophospholipid composition towards shorter acyl chains, compensatory increases in membrane rigidity, and dysregulation of virulence-associated lipid classes including phthiocerol dimycocerosates and triacylglycerols (Zabost et al. 2026). LC-MS-based lipidomic profiling of *Leishmania donovani*-infected macrophages quantitatively defined infection-driven changes across multiple lipid classes, revealing reductions in sphingomyelin and ceramide species, alongside a broad downregulation of phospholipid subclasses, providing mechanistic insight into pathogen-induced changes in membrane fluidity and antigen presentation (Tabrez et al. 2024).

For T4SS pathogens, equivalent comprehensive lipidomics remains largely absent. Closest to a lipidomic approach applied to a T4SS pathogen, Clemente and colleagues performed quantitative LC-MS/MS sterol profiling of sorted *Coxiella*-infected macrophages, quantifying 13 free sterol species and identifying selective accumulation of 25-hydroxycholesterol in cells infected with an Stmp1 mutant—revealing that *Coxiella*’s own sterol-modifying enzyme actively suppresses this oxysterol to avoid its effect on CCV integrity and bacterial replication (Clemente et al. 2022). The recent systematic identification of over 30 novel PI-binding *Legionella* effectors using lipid bead pulldowns coupled to mass spectrometry (Bolt 2025) represents a step towards higher-throughput lipid-focused analyses, though this approach interrogates effector/lipid interactions rather than the host lipidome per se.

Looking ahead, several applications stand out as particularly tractable. Lipidomic profiling of purified bacteria-containing vacuoles, made feasible by existing density gradient enrichment protocols for LCVs and CCVs, would provide the first comprehensive lipid maps of these compartments, moving beyond individual lipid markers towards a systems-level understanding of vacuole identity. Temporal lipidomics across the infection cycle could capture the ordered sequence of lipid transitions that accompany vacuole maturation, potentially identifying rate-limiting remodelling events that are invisible to targeted approaches. Finally, integration of lipidomics with CRISPR-based genetic screens offers a path to systematically assign host lipid pathways to effector function, offering the possibility of moving from the current effector-by-effector characterization of lipid dependencies towards a systems-level map of host lipid pathway requirements during infection.

## Conclusion

The study of T4SS-equipped intracellular bacteria, including *L. pneumophila, C. burnetii, B. abortus*, and *Anaplasma/Ehrlichia* species, arrived at a convergent insight: controlling the lipid composition of the host membrane is equivalent to controlling the host cell itself. By rewriting the phosphoinositide code on their vacuolar membranes, recruiting host lipid-transfer proteins, tapping into lipid droplets for energy, and modulating bioactive lipid mediators to suppress immunity, these pathogens construct intracellular niches of remarkable stability. The effector proteins through which these manipulations are executed constitute a structurally and mechanistically diverse arsenal, from the structurally distinct PI4P-binding modules of *Legionella* DrrA and SidC, to the TIRAP-mimicking architecture of *Brucella* BtpA, which recruits phosphoinositides through its N-terminal domain while its TIR domain subverts innate immune signalling.

Several convergent themes emerge from comparative analyses. PIs have emerged as key components of vacuoles harbouring *Legionella* and *Coxiella* (Weber et al. 2006, Martinez et al. 2016, Bienvenu et al. 2024), whereas for *Brucella* and *Anaplasma/Ehrlichia*, vacuolar phosphoinositide composition remains less well-characterized. Cholesterol manipulation is a recurrent strategy across these pathogens, whether through lipid raft-dependent host cell entry, well-established for *Brucella* (Naroeni and Porte 2002, Watarai et al. 2002, 2002, Arellano-Reynoso et al. 2005), *Coxiella* (Gilk et al. 2013), and *Anaplasma/Ehrlichia* (Lin and Rikihisa 2003), or through active regulation of vacuolar sterol content, as exemplified by *Coxiella*’s Stmp1 (Clemente et al. 2022). Lipid droplets function as nutrient reservoirs in *Coxiella* infection (Angara et al. 2024) and host fatty acid availability is a key determinant of *Legionella* replication vacuole integrity and bacterial growth (Ghosh et al. 2024). T4SS effectors across all four pathogens are notably enriched in lipid-binding modules, whether long-characterized PI-selective domains or the recently identified compact α-helical folds, supporting the view that membrane targeting is a fundamental architectural principle of T4SS effector design.

The divergences are equally informative. *Coxiella*’s tolerance of a phagolysosomal environment distinguishes it sharply from *Legionella* and *Brucella*, and the three effectors identified thus far reflect the need to sculpt a complex acidic vacuole rather than prevent its maturation along the endocytic pathway.

As lipidomics technology advances towards single-cell resolution, real-time lipid imaging, and deep multi-omics integration, the landscape of lipid-mediated host/pathogen interaction will come into ever sharper focus. The convergent strategies identified in this review are the visible surface of a much richer molecular ecology and mapping it fully, then intervening in it precisely, represents a promising frontier in the cell biology of infection. These convergent, lipid-centred dependencies also point toward host-directed therapeutic strategies: pharmacological correction of the cholesterol, phosphoinositide, or lipid-droplet pathways exploited by these pathogens, or direct inhibition of the effectors that hijack them, could in principle restore normal membrane trafficking and immune signalling independently of antibiotic susceptibility, offering a complementary route to combating infections caused by genetically intractable or drug-tolerant intracellular bacteria.
